# Effects of a 3-Week In-Hospital Body Weight Reduction Program on Cardiovascular Risk Factors, Muscle Performance, and Fatigue: A Retrospective Study in a Population of Obese Adults with or without Metabolic Syndrome

**DOI:** 10.3390/nu12051495

**Published:** 2020-05-21

**Authors:** Antonello E. Rigamonti, Sabrina Cicolini, Diana Caroli, Alessandra De Col, Massimo Scacchi, Silvano G. Cella, Alessandro Sartorio

**Affiliations:** 1 Department of Clinical Sciences and Community Health, University of Milan, 20129 Milan, Italy; massimo.scacchi@unimi.it (M.S.); silvano.cella@unimi.it (S.G.C.); 2 Experimental Laboratory for Auxo-endocrinological Research, Istituto Auxologico Italiano, IRCCS, 28824 Verbania, Italy; s.cicolini@auxologico.it (S.C.); d.caroli@auxologico.it (D.C.); a.decol@auxologico.it (A.D.C.); sartorio@auxologico.it (A.S.); 3 Division of General Medicine, Istituto Auxologico Italiano, IRCCS, 28824 Verbania, Italy; 4 Division of Auxology and Metabolic Diseases, Istituto Auxologico Italiano, IRCCS, 28824 Verbania, Italy

**Keywords:** body weight reduction program, obesity, metabolic syndrome, cardiovascular risk factors, stair climbing test, fatigue severity score, body mass index

## Abstract

Background. In clinical practice, there is the diffuse conviction that obese subjects with metabolic syndrome may be more difficult to treat. Objectives and Methods. The aim of the present study was that to investigate the effectiveness of a 3-week in-hospital body weight reduction program (BWRP) in a large population of obese subjects with and without metabolic syndrome (*n* = 1922; 222 men and 1700 women, age range 18–83 yr). Outcomes such as body mass index (BMI), total (TOT) and HDL cholesterol, systolic and diastolic blood pressures (SBP and DBP, respectively), coronary heart disease (CHD) score, fatigue severity score (FSS), and stair climbing test (SCT) time were evaluated before and after the intervention (Δ). A sex-, BMI-, and age-related stratification of the obese population with or without metabolic syndrome was applied. Results. When compared to obese subjects without metabolic syndrome, at the basal conditions, obese subjects had a poorer cardiometabolic profile, as demonstrated by higher triglycerides, TOT-cholesterol, DBP, SBP, and CHD score, and a more compromised muscle performance (evaluated by SCT), associated with more perception of fatigue (measured by FSS). Nevertheless, obese subjects with metabolic syndrome obtained more benefits from BWRP than those without metabolic syndrome for some outcomes (i.e., ΔTOT-cholesterol, ΔSBP, and ΔCHD score). Despite these differences, the BWRP-induced weight loss was similar between the two groups (i.e., ΔBMI) as well as the gain of muscle performance (i.e., ΔSCT) and the reduction of fatigue (i.e., ΔFSS). Interestingly, the potentially deleterious fall in HDL-cholesterol levels after BWRP was less evident in obese subjects with metabolic syndrome than those without metabolic syndrome. When pooling all data, the ΔCHD score was associated with age, sex, and metabolic syndrome. The remaining outcomes, such as ΔBMI, ΔFSS, and ΔSCT time, were associated with sex and age but not with metabolic syndrome. Finally, ΔBMI was positively correlated with ΔCHD score, ΔFSS, and ΔSCT time in both obese subjects without metabolic syndrome and obese subjects with metabolic syndrome. Conclusions. When comparing obese subjects undergoing a BWRP, metabolic syndrome is not a negative predictive factor affecting the effectiveness of this intervention in terms of weight loss, muscle performance, and psychological well-being.

## 1. Introduction

Metabolic syndrome is a clinically and epidemiologically relevant condition that identifies obese subjects having high coronary heart disease (CHD) risk due to the coexistence of abdominal adiposity, dyslipidemia, blood hypertension, and glucose intolerance or diabetes mellitus [1].

Although pharmacological research is actively seeking new anti-obesity strategies [2,3,4,5], restricted energy intake, combined to moderate aerobic physical activity, represents the most effective treatment of obesity associated with metabolic syndrome, despite that weight loss may be modest over a short-term cycle of treatment and weight regain may rapidly compromise the enormous efforts made to reduce body weight [6]. In fact, the beneficial effects associated with weight loss, even modest weight loss, are manifold, including reduction of total cholesterol and blood pressure, particularly systolic blood pressure, improvement of gluco-metabolic homeostasis, attenuation of the low-grade chronic inflammation, increase in motor control/function and psychological well-being as documented by the perception of fatigue and quality of life [7,8,9,10,11,12]. Being that dyslipidemia, blood hypertension, and diabetes mellitus are some of the most relevant CHD risk factors, weight loss results in a reduction of CHD score [13].

Nevertheless, in clinical practice, there is the diffuse opinion that obese subjects with metabolic syndrome are more difficult to treat because of the excessive abdominal adiposity and the seriously compromised cardiometabolic profile [14]. Indeed, while many clinical studies have investigated the effects of different body weight reduction programs (BWRPs) in obese subjects with metabolic syndrome on specific CHD-related outcomes [15,16], to the best of our knowledge, no author has compared the effectiveness of such an intervention in a population of obese patients with and without metabolic syndrome.

So, the aim of the present study was that of evaluating the effects of a BWRP, consisting in restricted energy intake, physical rehabilitation (i.e., moderate aerobic activity), psychological counseling, and nutritional education, in a large population of obese adults of both sexes, with or without metabolic syndrome. Outcomes such as weight loss, total (TOT-) and high-density lipoprotein (HDL-) cholesterol, diastolic and systolic blood pressures (DBP and SBP, respectively), CHD score, fatigue severity score (FSS), and stair climbing test (SCT) time were evaluated before and after BWRP and compared between obese subjects with and without metabolic syndrome. Furthermore, the two groups were stratified on the basis of sex, body mass index (BMI), and age, which are known to impact the success of any BWRP [8,17,18,19,20].

Our hypothesis is that metabolic syndrome does not represent a negative predictive factor for any obese subject that is willing to lose weight and obtain BWRP-induced benefits, such as reduction of CHD score, improvement of muscle performance, and psychological well-being.

## 2. Materials and Methods

### 2.1. Subjects

A set of 1992 adults were retrospectively selected from the total patient population admitted to the Division of Metabolic Diseases of the Istituto Auxologico Italiano, Piancavallo (VB), Italy, between March 2015 and January 2020.

The inclusion criteria were individuals of both sexes, older than 18 years, having a BMI >30 kg/m^2^, with or without metabolic syndrome (according to IDF, International Diabetes Federation) [21], independently of the level of physical activity. The exclusion criteria were (1) individuals with systolic blood pressure (SBP) ≥180 mmHg and diastolic blood pressure (DBP) ≥110 mmHg; (2) cardiovascular disease clinically evident in the past 6 months manifested by myocardial infarction or stroke; (3) neurological, muscular, or rheumatologic diseases that limit the ability to undertake the tests of the protocol (see below); (4) disabling clinical symptoms reported during the SCT test; (5) individuals who refused to sign the consent form.

After clinical evaluation, based on the above-reported inclusion/exclusion criteria, 29 patients with a history of cardiovascular diseases (including acute myocardial infarction, chronic heart failure, or angina pectoris) were excluded from the study; furthermore, there was a drop-out of 41 patients due to spontaneous withdrawal from the study (occurring after a mean of 12 ± 2.5 days from the beginning of the BWRP), leading to a total of 1922 patients to be recruited and included in the statistical analysis (222 men and 1700 women, age range 18–83 yr). Of these, 336 (17.5%) were taking medications for diabetes, 1034 (53.8%) for hypertension, and 153 (8%) for dyslipidemia. Medical treatments for concomitant diseases were continued throughout the study.

The protocol was approved by the Ethical Committee of the Istituto Auxologico Italiano (research project code: 18A301; acronym: FUOBAUXO); the protocol was explained to the patients, who gave their written informed consent.

Before and after BWRP, each subject underwent the following tests/evaluations, which are described in details below:

- CHD score;

- standard hematochemistry;

- stair climbing test (SCT);

- fatigue severity score (FSS).

### 2.2. Body Weight Reduction Program (BWRP)

The BWRP consisted of a 3-week in-hospital integrated energy-restricted diet (1200–1800 kcal/day) in combination with physical rehabilitation (moderate aerobic activity), psychological counseling, and nutritional education. The amount of energy to be given with diet was calculated by subtracting approximately 500 kcal from the measurement of resting energy expenditure. The diet, in terms of macronutrients, contained 21% proteins, 53% carbohydrates, and 26% lipids; the daily estimated water content was 1000 mL, while the estimated salt content was 1560 mg Na^+^, 3600 mg K^+^, and 900 mg Ca^+2^. Extra water intake of at least 2000 mL/day was encouraged.

The physical activity program consisted of 5 days *per* week training, including (i) 1 h dynamic aerobic standing and floor exercise with arms and legs, at moderate intensity and under the guidance of a therapist; and (ii) either 20–30 min cycloergometer exercise at 60 W, or 3–4 km out-door walking on flat terrain, according to individual capabilities and clinical status. 

The subjects also underwent a psychological counseling program consisting of two or three sessions per week of individual and/or group psychotherapy performed by clinical psychologists. Furthermore, lectures, demonstrations, and group discussions, with or without a supervisor, took place daily.

### 2.3. Evaluation of Risk Factors

Selected CHD risk factors, including DBP and SBP, TOT- and HDL-cholesterol, cigarette smoking, and diabetes [22], were assessed at hospital admission and on the last day of the 3-week BWRP. 

The medical history was taken, and a physical examination was performed. Height and weight were measured, respectively, with a Harpenden stadiometer (Holtain Ltd., UK), and an electronic scale (Selus, Italy) BMI (kg/m^2^) was then calculated. Two BP determinations were made after the participants had been sitting at least 5 min, and the mean value was used for the statistical analysis.

Blood samples were collected after an overnight fast in standard tubes for serum. TOT-cholesterol, HDL-cholesterol, triglycerides, and glucose levels were immediately measured with enzymatic-colorimetric methods (Hitachi Instrument, Japan). The intra- and inter-assay coefficients of variation (CV) of each analytical procedure were the following values: 1.1% and 1.6% (TOT-cholesterol), 1.8% and 2.2% (HDL-cholesterol), 1.1% and 2.0% (triglycerides), and 1.0% and 1.3% (glucose). While TOT- and HDL-cholesterol levels were measured before and after BWRP, triglycerides and glucose levels were measured only before the BWRP, since they were necessary for the diagnosis of metabolic syndrome and diabetes mellitus.

Blood pressure and TOT- or HDL-cholesterol and triglyceride levels were considered without regard to the use of antihypertensive or lipid-lowering medications. 

Diabetes was considered present if the patient was under treatment with insulin or oral hypoglycemic agents, or if fasting blood glucose levels exceeded 140 mg/dL at the initial examination.

Persons who smoked at least one cigarette per day during the previous 12 months were classified as smokers. For this reason, although the smokers were not allowed to smoke during the short hospitalization period, they were considered as smokers through the entire BWRP. 

The CHD scores were estimated using a simple coronary disease prediction model developed by Wilson et al. [22], which takes into account sex, age, diabetes, smoking, BP, and TOT- and HDL-cholesterol categories.

These variables have been demonstrated to be independent and biologically important risk factors for CHD. Additionally, this score has the advantage of providing a simplified approach to predict risk for initial CHD events in disease-free out-patients.

CHD score sheets (for men and women) attribute different ranks in the function of classes of age (nine subgroups, from 30 up to 74 yr), TOT-cholesterol (five subgroups, from <160 to ≥280 mg/dL), HDL-cholesterol (five subgroups, from <35 up to ≥60 mg/dL) and BP (SBP: five subgroups, from <120 up to ≥160 mmHg; DBP: five subgroups, from <80 up to ≥100 mmHg). Diabetes and smoking were defined into two categories (yes/no).

Although SBP and DBP values were used, when SBP and DBP fell into different categories, the higher category was selected for the calculation of the CHD score.

### 2.4. Stair Climbing Test 

SCT is a well-standardized procedure that was readapted and validated by our group to measure maximal anaerobic power [23,24,25,26]. Before the execution of the test, 2–3 practice trials were allowed so that subjects could become adequately confident with the technique. Briefly, subjects were invited to climb up ordinary stairs at the highest possible speed, according to their capabilities. The stairs consisted of 13 steps of 15.3 cm each, thus covering a total vertical distance of 1.99 mt. An experimental investigator measured the time taken to perform the test by using a digital stopwatch. 

### 2.5. Fatigue Severity Scale

FSS is one of the self-report questionnaires most commonly used to measure fatigue in patients with chronic diseases. FSS encompasses the physical, social, and cognitive effects of fatigue [27,28], and has already been used and validated in obese Italian patients by our group [24]. 

FSS consists of 9 statements (items) describing the negative effects of fatigue on motivation, exercise, physical functioning, ability to carry out duties, work, family or social life. Responders are asked to rate each statement considering the previous week and using a Likert scale ranging from 1 (strong disagreement) to 7 (strong agreement). The total score is computed by averaging the raw scores of each item. 

### 2.6. Statistical Analysis

Sigma Stat 3.5 statistical software package (Systat Software, San Jose, CA, USA) was used for data analyses, and GraphPad Prisma 5.0 software (GraphPad Software, San Diego, CA, USA) was used for data plotting.

Results were reported as mean ± SD (standard deviation). Each parameter, particularly BMI, TOT-cholesterol, HDL-cholesterol, DBP, SBP, CHD score, FSS, and SCT time (outcomes), were evaluated not only as continuous variables but also as percent pre–post-BWRP difference (Δ as %). In figures, the negative or positive values were maintained; to facilitate the description and understanding of the results (i.e., in the text), if the pre–post-BWRP difference itself was negative (Δ < 0), then the corresponding positive value was considered (|Δ| = −Δ > 0). 

The sample size was calculated, supposing that ΔBMI and ΔFSS were correlated with an r-value of 0.07 in a test at a power of 80% and an α error of 0.05 (*n* = 1600). FSS was chosen for this calculation because it is the most subjective among the remaining outcomes.

Before applying any parametric test, the normalcy of data distribution was verified by using the Shapiro–Wilk test. Being the main subdivision of the recruited population in subjects with or without metabolic syndrome, all parameters were compared among sex-, age-, and BMI-based subgroups (all, females/males, obese subjects with BMI ≥40 kg/m^2^ (i.e., degree 3) or <40 kg/m^2^ (degrees 1–2), respectively, and older/non-older for ≥65 years or <65 years, respectively) before and/or after BWRP by using a Student’s *t*-test for pair/unpair data or a one-way/two-way ANOVA, followed by posthoc Bonferroni’s test, when appropriate. When using a two-way ANOVA, apart from the status of metabolic syndrome, the second factor was sex, BMI, or age.

Coefficient of Pearson was calculated to correlate ΔBMI with ΔCHD score, ΔFSS, or ΔSCT time in all subjects with or without metabolic syndrome.

A model of multiple linear regression was used to predict ΔBMI, ΔCHD score, ΔFSS or ΔSCT time (as dependent variables) starting from the following independent variables: age, sex (F/M), pre-BWRP BMI, and metabolic syndrome (yes/no).

A level of significance of *p* < 0.05 was used for all the above-reported analyses.

## 3. Results

### 3.1. Total Population

When considering all population independently from sex, age, and BMI, obese subjects with metabolic syndrome had significantly higher age, BMI, levels of TOT-cholesterol, glucose, and triglycerides, DBP, SBP, CHD score, FSS, and SCT time than those without metabolic syndrome, while HDL-cholesterol levels were significantly lower (*p* < 0.05; Table 1).

BWRP significantly reduced BMI, levels of TOT- and HDL-cholesterol, DBP, SBP, FSS, and SCT time in both groups (*p* < 0.05). While CHD score was unchanged in obese subjects without metabolic syndrome, BWRP significantly reduced CHD score in obese subjects with metabolic syndrome (*p* < 0.05). Despite a similar weight loss between the two groups, the positive effects of BWRP in terms of ΔTOT-cholesterol, ΔDBP, ΔSBP, and ΔCHD score were significantly greater in obese subjects with metabolic syndrome than those without metabolic syndrome (*p* < 0.05). Furthermore, ΔHDL-cholesterol in obese subjects with metabolic syndrome after BWRP was significantly lower when compared to that in obese subjects without metabolic syndrome (*p* < 0.05).

### 3.2. Subdivision for Sex: Females and Males

When considering female population independently from age and BMI, obese females with metabolic syndrome had significantly higher age, BMI, levels of TOT-cholesterol, glucose, and triglycerides, DBP, SBP, CHD score, FSS, and SCT time than those without metabolic syndrome, whose levels of HDL-cholesterol were significantly higher (*p* < 0.05; Table 2).

BWRP significantly reduced BMI, levels of TOT- and HDL-cholesterol, DBP, SBP, FSS, and SCT time in both female groups (*p* < 0.05). While CHD score significantly increased in obese females without metabolic syndrome (*p* < 0.05), BWRP significantly reduced CHD score in obese females with metabolic syndrome (*p* < 0.05). 

When considering the male population independently from age and BMI, obese males with metabolic syndrome had significantly higher age, levels of TOT-cholesterol, and CHD score than those without metabolic syndrome, whose levels of HDL-cholesterol were significantly higher (*p* < 0.05; Table 2).

BWRP significantly reduced BMI, levels of TOT- and HDL-cholesterol, DBP, SBP, CHD score, FSS, and SCT time in both male groups (*p* < 0.05). 

**Figure 1:**
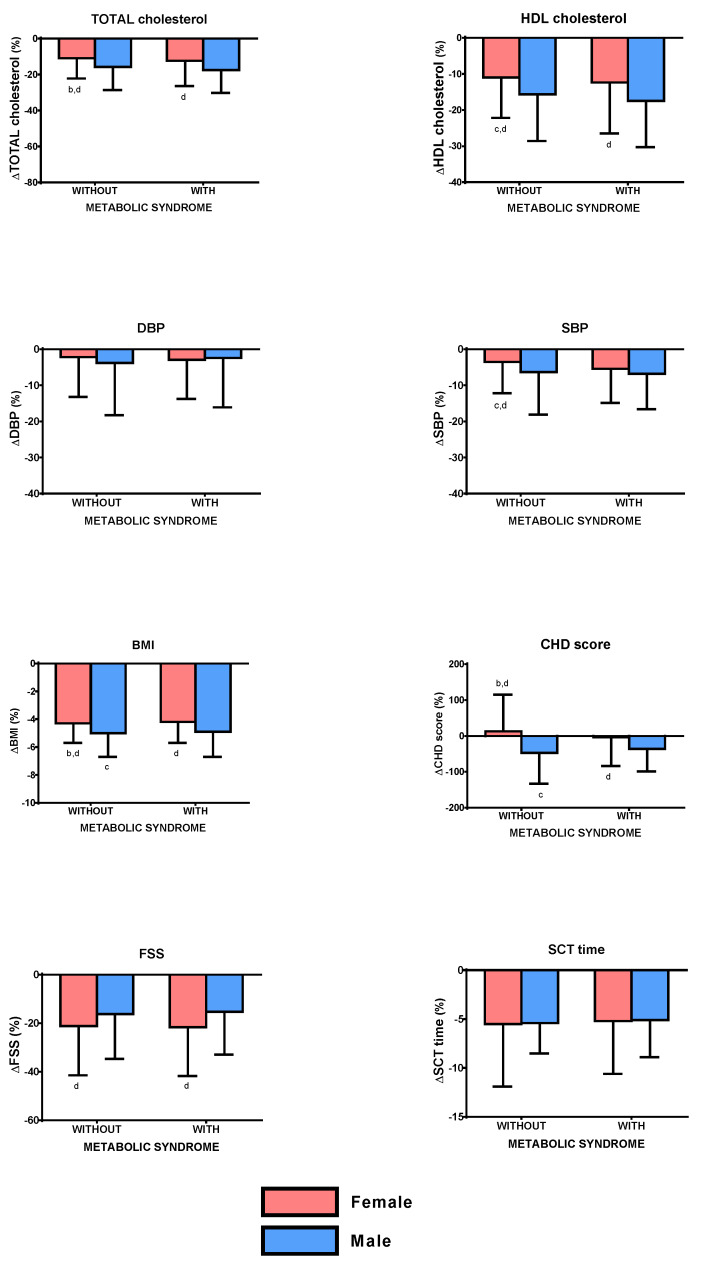
Changes of total cholesterol, HDL cholesterol, diastolic blood pressure (DBP), systolic blood pressure (SBP), body mass index (BMI), coronary heart disease (CHD) score, fatigue severity score (FSS), and stair-climbing test (SCT) time, before and after a three-week body weight reduction program (BWRP) in obese subjects with or without metabolic syndrome, subdivided for sex (females/males). Data are expressed as mean ± SD. All parameters were compared among all subgroups (females/males and with/without metabolic syndrome) by using a two-way ANOVA, followed by post hoc Bonferroni’s test. ^b^: 0.05 vs. obese males without metabolic syndrome; ^c^: *p* < 0.05 vs. obese females with metabolic syndrome; ^d^: *p* < 0.05 vs. obese males with metabolic syndrome. In order to facilitate the interpretation of multiple comparisons, please, note that the four boxes (1, 2, 3, and 4) of each panel may be named as “a”, “b”, “c”, and “d” from the left side to the right one. Therefore, when the symbol of significance is indicated under a box (e.g., “c” under box 2), this means that the comparison between the two boxes (i.e., boxes 2 and 3) is statistically significant.

BWRP induced a significantly higher ΔTOT-cholesterol in obese males than the corresponding female group with or without metabolic syndrome (*p* < 0.05). Moreover, obese females without metabolic syndrome had a significantly lower ΔTOT-cholesterol than obese males with metabolic syndrome (*p* < 0.05; Figure 1; to more easily understand the comparisons, carefully read the footnotes).

BWRP induced a significantly lower ΔHDL-cholesterol in obese females with metabolic syndrome than the corresponding male group (*p* < 0.05). Moreover, obese females without metabolic syndrome had significantly lower ΔHDL-cholesterol than female or male groups with metabolic syndrome (*p* < 0.05).

Differently from ΔDBP, which was similar in all groups (females/males and with/without metabolic syndrome), BWRP induced a significantly lower ΔSBP in obese females without metabolic syndrome than both female and male groups with metabolic syndrome (*p* < 0.05).

BWRP induced a significantly lower ΔBMI in obese females with or without metabolic syndrome than the corresponding male group (*p* < 0.05). Moreover, obese males without metabolic syndrome had a significantly higher ΔBMI than obese females with metabolic syndrome (*p* < 0.05), while obese females without metabolic syndrome had a significantly lower ΔBMI than obese males with metabolic syndrome (*p* < 0.05).

BWRP induced a significantly lower ΔCHD score in obese females with or without metabolic syndrome than the corresponding male group (*p* < 0.05). Moreover, obese males without metabolic syndrome had a significantly higher ΔCHD score than obese females with metabolic syndrome (*p* < 0.05), while obese females had a significantly lower ΔCHD score than obese males with metabolic syndrome (*p* < 0.05).

Differently from ΔSCT time, which was similar in all groups (females/males and with/without metabolic syndrome), BWRP induced a significantly lower ΔFSS in obese males with metabolic syndrome than obese females with or without metabolic syndrome (*p* < 0.05).

### 3.3. Subdivision for BMI: <40 and ≥40 kg/m^2^

When considering 1–2-degree obese population, i.e., that having a BMI <40 kg/m^2^, independently from age and sex, obese subjects with metabolic syndrome had significantly higher age, levels of TOT-cholesterol, glucose and triglycerides, DBP, SBP, CHD score, FSS, and SCT time than those without metabolic syndrome, while HDL-cholesterol levels were significantly lower (*p* < 0.05; Table 3).

BWRP significantly reduced BMI, levels of TOT- and HDL-cholesterol, DBP, SBP, FSS, and SCT time in both 1–2-degree obese groups (*p* < 0.05); while CHD score was unchanged in obese subjects without metabolic syndrome (*p* < 0.05), BWRP significantly reduced CHD score in those with metabolic syndrome (*p* < 0.05). 

When considering 3-degree obese population, i.e., that having a BMI ≥40 kg/m^2^, independently from age and sex, obese subjects with metabolic syndrome had significantly higher age, levels of TOT-cholesterol and CHD score than those without metabolic syndrome, while HDL-cholesterol levels were significantly lower (*p* < 0.05; Table 3).

BWRP significantly reduced BMI, levels of TOT- and HDL-cholesterol, DBP, SBP, FSS, and SCT time in both 3-degree obese groups (*p* < 0.05); while CHD score was unchanged in obese subjects without metabolic syndrome (*p* < 0.05), BWRP significantly reduced CHD score in obese subjects with metabolic syndrome (*p* < 0.05).

**Figure 2:**
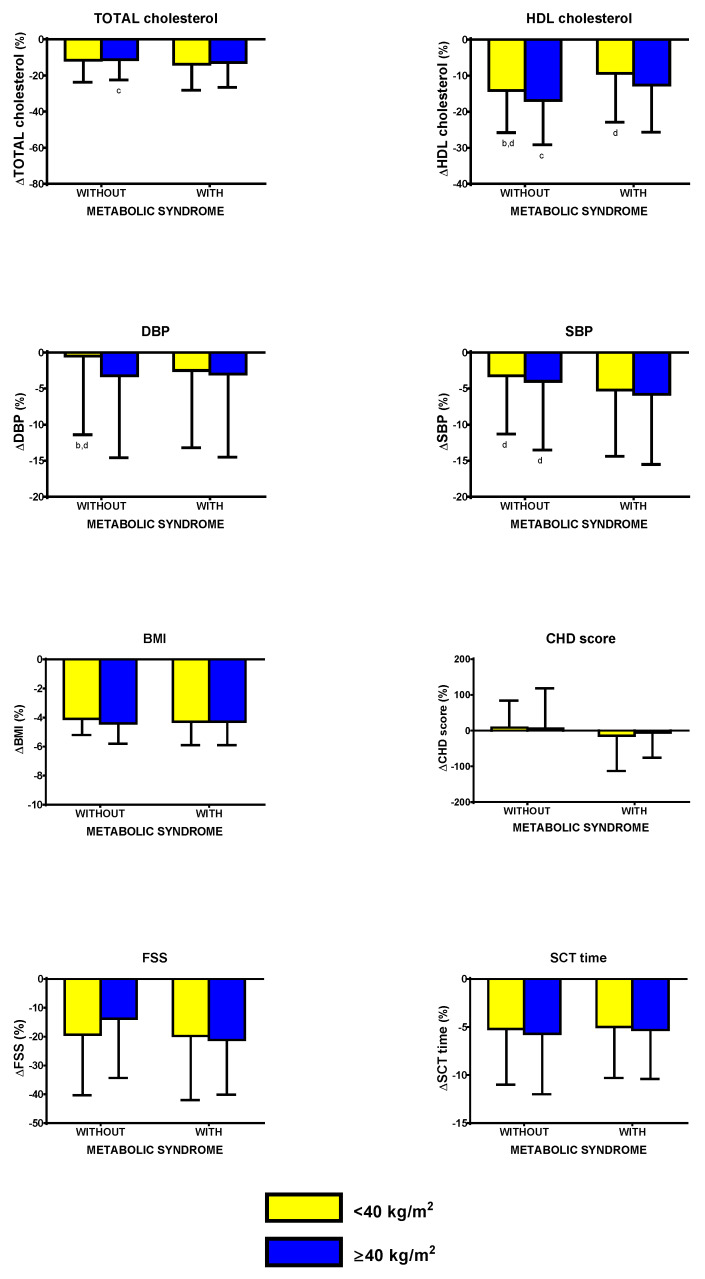
Changes of total cholesterol, HDL cholesterol, diastolic blood pressure (DBP), systolic blood pressure (SBP), body mass index (BMI), coronary heart disease (CHD) score, fatigue severity score (FSS), and stair climbing test (SCT) time, before and after a three-week body weight reduction program (BWRP) in obese subjects with or without metabolic syndrome, subdivided for BMI (<40 kg/m^2^ or obese subjects of degree 1–2 and ≥40 kg/m^2^ or obese subjects of degree 3). Data are expressed as mean ± SD. All parameters were compared among all subgroups (</≥40 kg/m^2^ and with/without metabolic syndrome) by using a two-way ANOVA, followed by post hoc Bonferroni’s test. ^b^: 0.05 vs. <40 kg/m^2^ obese subjects without metabolic syndrome; ^c^: *p* < 0.05 vs. ≥40 kg/m^2^ obese subjects with metabolic syndrome; ^d^: *p* < 0.05 vs. ≥40 kg/m^2^ obese subjects with metabolic syndrome. In order to facilitate the interpretation of multiple comparisons, please, note that the four boxes (1, 2, 3, and 4) of each panel may be named as “a”, “b”, “c”, and “d” from the left side to the right one. Therefore, when the symbol of significance is indicated under a box (e.g., “c” under box 2), this means that the comparison between the two boxes (i.e., boxes 2 and 3) is statistically significant.

BWRP induced a significantly lower ΔTOT-cholesterol in 3-degree obese subjects without metabolic syndrome than in those with metabolic syndrome (*p* < 0.05; Figure 2; to more easily understand the comparisons, carefully read the footnotes).

BWRP induced a significantly lower ΔHDL-cholesterol in 1–2-degree obese subjects with and without metabolic syndrome than the corresponding 3-degree obese group (*p* < 0.05). Moreover, 1–2-degree obese subjects without metabolic syndrome had a significantly higher ΔHDL-cholesterol than 3-degree obese subjects with metabolic syndrome (*p* < 0.05), while 3-degree obese subjects without metabolic syndrome had a significantly higher ΔHDL-cholesterol than 1–2-degree obese subjects with metabolic syndrome (*p* < 0.05).

BWRP induced a significantly lower ΔDBP in 1–2-degree obese subjects without metabolic syndrome than 3-degree obese subjects with and without metabolic syndrome (*p* < 0.05).

BWRP induced a significantly lower ΔSBP in 1–2-degree obese subjects without metabolic syndrome than in 3-degree obese subjects with metabolic syndrome (*p* < 0.05).

BWRP induced no significant differences in values of ΔBMI, ΔCHD score, ΔFSS, and ΔSCT time among all groups (1–2-degree/3-degree obese subjects, with/without metabolic syndrome).

### 3.4. Subdivision for Age: <65 and ≥65 yr

When considering non-older obese population, i.e., those aged <65 yr, independently from BMI and sex, non-older obese subjects with metabolic syndrome had significantly higher age, levels of TOT-cholesterol, glucose and triglycerides, DBP, SBP, CHD score, FSS, and SCT time than those without metabolic syndrome, whose levels of HDL-cholesterol were significantly higher (*p* < 0.05; Table 4).

BWRP significantly reduced BMI, levels of TOT- and HDL-cholesterol, DBP, SBP, FSS, and SCT time in both non-older obese groups (*p* < 0.05); while the CHD score was unchanged in non-older obese subjects without metabolic syndrome (*p* < 0.05), BWRP significantly reduced CHD score in non-older obese subjects with metabolic syndrome (*p* < 0.05).

When considering the older obese population, i.e., those aged ≥65 yr, independently from BMI and sex, older obese subjects with metabolic syndrome had significantly higher CHD scores than those without metabolic syndrome, whose levels of HDL-cholesterol were significantly higher (*p* < 0.05; Table 4).

Differently from the CHD score, which was unchanged, BWRP significantly reduced BMI, levels of TOT- and HDL-cholesterol, DBP, SBP, FSS, and SCT time in both older obese groups (*p* < 0.05).

**Figure 3:**
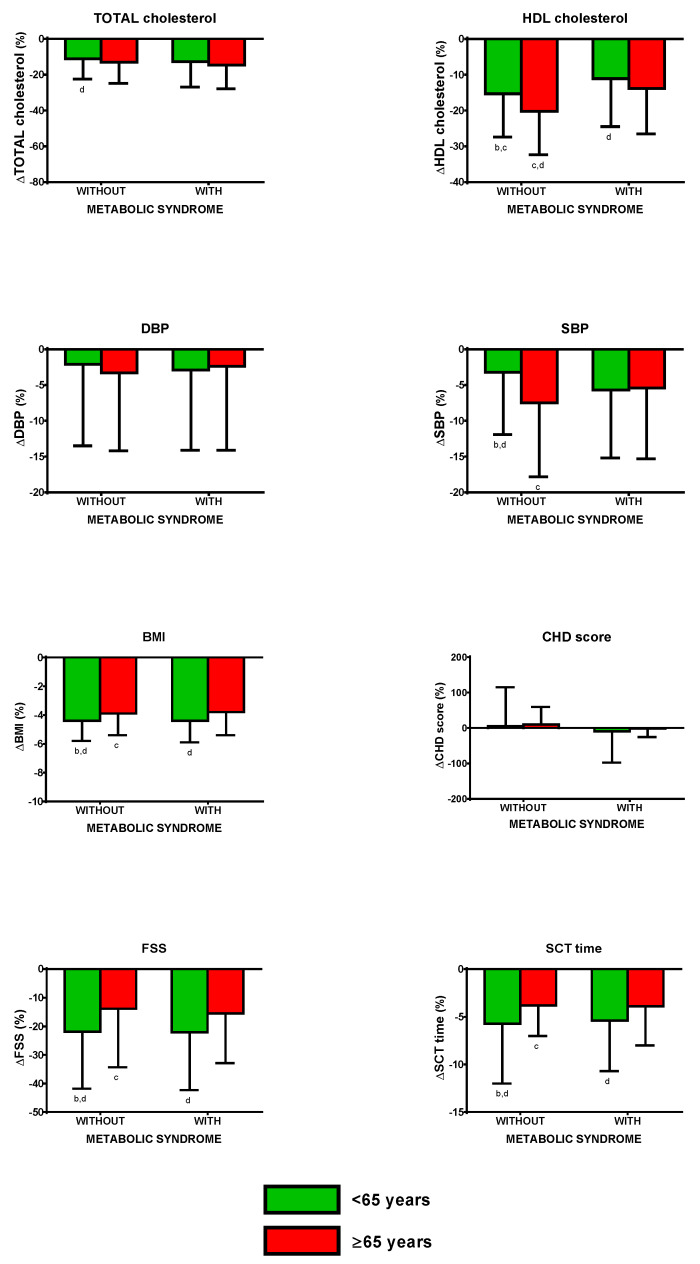
Changes of total cholesterol, HDL cholesterol, diastolic blood pressure (DBP), systolic blood pressure (SBP), body mass index (BMI), coronary heart disease (CHD) score, fatigue severity score (FSS), and stair climbing test (SCT) time, before and after a three-week body weight reduction program (BWRP) in obese subjects with or without metabolic syndrome, subdivided for age (<65 years or non-older obese subject and ≥65 years or older obese subject). Data are expressed as mean ± SD. All parameters were compared among all subgroups (</≥65 years and with/without metabolic syndrome) by using a two-way ANOVA, followed by post hoc Bonferroni’s test. ^b^: 0.05 vs. <65 years old obese subjects without metabolic syndrome; ^c^: *p* < 0.05 vs. ≥65 years old obese subjects with metabolic syndrome; ^d^: *p* < 0.05 vs. ≥65 years old obese subjects with metabolic syndrome. In order to facilitate the interpretation of multiple comparisons, please, note that the four boxes (1, 2, 3, and 4) of each panel may be named as “a”, “b”, “c”, and “d” from the left side to the right one. Therefore, when the symbol of significance is indicated under a box (e.g., “c” under box 2), this means that the comparison between the two boxes (i.e., boxes 2 and 3) is statistically significant.

BWRP induced a significantly lower ΔTOT-cholesterol in non-older obese subjects without metabolic syndrome than older obese subjects with metabolic syndrome (*p* < 0.05; Figure 3; to more easily understand the comparisons, carefully read the footnotes).

BWRP induced a significantly lower ΔHDL-cholesterol in non-older obese subjects with and without metabolic syndrome than the corresponding older obese group (*p* < 0.05).Moreover, non-older obese subjects with metabolic syndrome had a significantly lower ΔHDL-cholesterol than non-older and older obese subjects without metabolic syndrome (*p* < 0.05), while older obese subjects without metabolic syndrome had a significantly higher ΔHDL-cholesterol than older obese subjects with metabolic syndrome (*p* < 0.05).

Differently from ΔDBP, which was similar in all groups (non-older/older obese subjects and with/without metabolic syndrome), BWRP induced a significantly lower ΔSBP in non-older obese without metabolic syndrome than older obese subjects with and without metabolic syndrome (*p* < 0.05). Moreover, the BWRP induced a significantly higher ΔSBP in older obese subjects without metabolic syndrome than non-older obese subjects with metabolic syndrome.

BWRP induced a significantly higher ΔBMI in non-older obese subjects with or without metabolic syndrome than the corresponding older group (*p* < 0.05). Moreover, non-older obese subjects without metabolic syndrome had a significantly lower ΔBMI than older obese subjects with metabolic syndrome (*p* < 0.05), while older obese subjects without metabolic syndrome had a significantly lower ΔBMI than older obese subjects with metabolic syndrome (*p* < 0.05).

Differently from ΔCHD score, which was similar in all groups (non-older/older obese subjects and with/without metabolic syndrome), BWRP induced a significantly higher ΔFSS in non-older obese subjects with or without metabolic syndrome than the corresponding older group (*p* < 0.05). Moreover, non-older obese subjects without metabolic syndrome had a significantly higher ΔFSS than older obese subjects with metabolic syndrome (*p* < 0.05), while older obese subjects without metabolic syndrome had a significantly lower ΔFSS than non-older obese subjects with metabolic syndrome (*p* < 0.05).

BWRP induced a significantly higher ΔSCT time in non-older obese subjects with or without metabolic syndrome than the corresponding older group (*p* < 0.05). Moreover, non-older obese subjects without metabolic syndrome had a significantly higher ΔSCT time than older obese subjects with metabolic syndrome (*p* < 0.05), while older obese subjects without metabolic syndrome had a significantly lower ΔSCT time than non-older obese subjects with metabolic syndrome (*p* < 0.05).

### 3.5. Regressions and Correlations

Analyzing all data in a model of multiple linear regression, including age, BMI, and sex as independent variables, ΔBMI as a dependent variable was significantly predicted by age (β = 0.195; *p* < 0.001) and sex (β = −0.124; *p* < 0.001, but not by pre-BWRP BMI (β = −0.007; *p* = 0.804) and metabolic syndrome (β = −0.002; *p* = 0.921). Age (β = 0.117; *p* < 0.001) and sex (β = −0.138; *p* < 0.001) as independent variables significantly predicted ΔSCT as a dependent variable, the contributions of the remaining independent variables pre-BWRP BMI (β = −0.021; *p* = 0.079) and metabolic syndrome (β = −0.049; *p* = 0.123) being not significant. ΔFSS as a dependent variable was significantly predicted by age (β = 0.074; *p* = 0.013) and sex (β = 0.116; *p* < 0.001), but not by the remaining independent variables pre-BWRP BMI (β = −0.006; *p* = 0.819) and metabolic syndrome (β = −0.019; *p* = 0.524). Finally, age (β = 0.156; *p* < 0.001), sex (β = −0.127; *p* < 0.001), and metabolic syndrome (β = −0.154; *p* < 0.001) as independent variables significantly predicted ΔCHD score as a dependent variable, the contribution of pre-BWRP BMI (β = 0.041; *p* = 0.185) being not significant.

**Figure 4:**
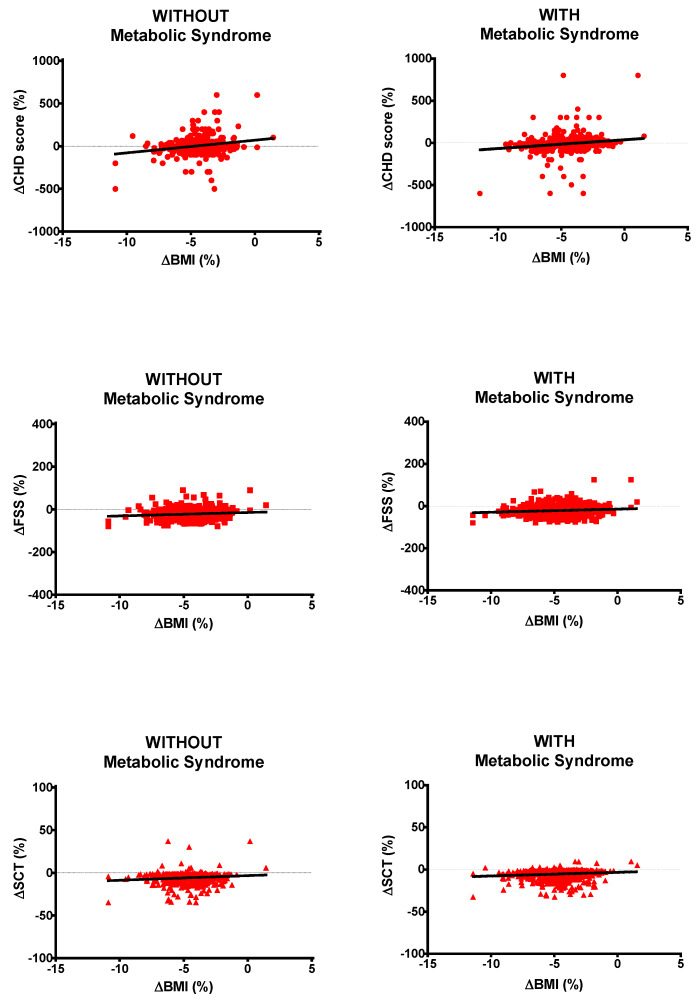
Correlations of ΔBMI (body mass index) with ΔCHD (coronary heart disease) score, ΔFSS (fatigue severity score) and ΔSCT (stair-climbing test) time for all obese subjects without (left panels) or with (right panels) metabolic syndrome. Please, note that Δ means pre–post-BWRP percent change.

Analyzing data from all obese subjects with or without metabolic syndrome, ΔBMI was positively correlated with ΔCHD score (*r* = 0.187 and *p* < 0.05 or *r* = 0.218 and *p* < 0.05, respectively), ΔFSS (*r* = 0.116 and *p* < 0.05 or *r* = 0.115 and *p* < 0.05, respectively) and ΔSCT (*r* = 0.129 and *p* < 0.05 or *r* = 0.124 and *p* < 0.05, respectively; Figure 4).

## 4. Discussion

The huge number of obese subjects recruited in the present study has allowed us to verify whether the status of metabolic syndrome may negatively affect the effectiveness of a short-term standardized in-hospital BWRP, entailing an energy-restricted diet, physical rehabilitation (moderate aerobic activity), psychological counseling and nutritional education, in specific sex-, BMI- and age-related subpopulations (i.e., females vs. males, <40 vs. ≥40 kg/m^2^, and <65 vs. ≥65 years, respectively).

Overall, based on the results obtained in the present study, we can argue that the occurrence of metabolic syndrome in the patient does not represent a factor limiting the therapeutic success of a short-term (i.e., 3 weeks) in-hospital BWRP. Paradoxically, despite that ΔBMI is equal between the two groups, obese subjects with metabolic syndrome obtain a considerable reduction of CHD score when compared to the counterpart without metabolic syndrome. This is presumably due to the more evident improvement of the cardiometabolic profile in the former than the latter ones, particularly reductions of TOT-cholesterol and SBP, which are some of the risk factors used to calculate CHD scores [22].

If simply admitting that the obese subjects with metabolic syndrome are cardiometabolically more compromised than the ones without metabolic syndrome, and tgus therapeutically more responsive, is not a fully convincing reason, then the apparently better responsiveness of the obese subject with metabolic syndrome to the positive effects of BWRP is difficult to explain. We can only suggest some putative mechanisms.

For instance, our BWRP is supposed to reduce the low-grade chronic inflammatory state that is known to characterize obesity, predominantly when associated with metabolic syndrome [29]. In this regard, some cytokines released by abdominal adipose tissue, which is dramatically expanded in obese subjects with metabolic syndrome, derange hepatic lipogenesis and glucometabolic homeostasis [30], and a regimen of restricted energy intake has been demonstrated to reduce some inflammatory markers, with an improvement of the cardiometabolic profile [29].

Furthermore, moderate and regular physical activity has been reported to reduce (resting) SBP because of hemodynamic adaptations, but also due to vasodilating factors such as nitric oxide (NO) and prostacyclin released by endothelium, which responds differently in a metabolically altered milieu such as that present in metabolic syndrome [31,32].

Obese subjects usually prefer “obesiogenic” foods, characterized by a high glycemic index, capable of promoting endothelial inflammation and dysfunction, whose deleterious effects occur already within a few hours after their ingestion [33]. Therefore, it is not surprising that the introduction of a “healthy” diet, such as that included in our BWRP, can dramatically improve the cardiometabolic status in obese subjects after a few weeks, particularly those with metabolic syndrome.

Fatigue is a frequently complained symptom in obesity and appears to be worse in metabolic syndrome, which is characterized by extreme adiposity and obesity-related sarcopenia [8,24,34,35,36,37]. In the present study, BWRP-induced changes in fatigue, measured by adopting FSS, an internationally validated questionnaire used in different pathophysiological contexts, including obesity [12,38], were similar between obese subjects with and without metabolic syndrome. As post-BWRP BMI was still higher in obese subjects with metabolic syndrome than those without metabolic syndrome, one can argue that the beneficial effect of BWRP on FSS does not depend uniquely upon the weight loss per se (e.g., gain of more agility in movements or improvement of sarcopenia), but may be a consequence of the “anti-fatiguing” effects of exercise at the psychological level (e.g., modulation of dopaminergic and serotoninergic pathways in some CNS areas) [39,40,41].

Similarly, in the present study, the BWRP-induced improvement of muscle performance, evaluated by SCT time, a method that we have successfully used in other studies carried out in different obese populations [8,9,25,26], was independent of the status of metabolic syndrome (i.e., same ΔSCT times in obese subjects with or without metabolic syndrome).

Seeing that the (pre-post-BWRP) ΔBMI is similar between the two groups, and having obese subjects with metabolic syndrome scored a higher post-BWRP SCT time than their counterparts without metabolic syndrome, weight loss and the ensuing agility of movement cannot be cited as unique mechanisms underlying the improvement of muscle performance in both groups.

We suppose an improvement of muscle biochemistry to be implied, particularly due to the reduction of the low-grade chronic inflammatory state after the completion of the BWRP [42]. In this regard, some cytokines have been demonstrated to be pathophysiologically involved in obesity-related sarcopenia and disturbances in energy production/utilization in muscle–skeletal cells, a mechanism that is predominant in obesity associated with metabolic syndrome [43,44]. Further studies are needed to demonstrate our hypothesis, starting from the measurement of circulating levels of some cytokines before and after a BWRP.

The above-reported positive results confirm and extend those obtained in another work of ours, which was carried out in an exclusively pediatric obese population with and without metabolic syndrome, who underwent a similar 3-week BWRP and were investigated for the same set of outcomes [9]. The immediate BWRP-induced benefits in obese children/adolescents with metabolic syndrome (in the previous study) might be less evident than those in obese adults with metabolic syndrome (in the present study), but, in a perspective view, the gain in terms of public health should be more important in the former group than the latter group.

The present study confirms the well-known resistance of obese females and older obese subjects to weight loss when undergoing a BWRP, and no further discussion of these findings is thought to be necessary [8,18]. However, the novelty is that obese females or older obese subjects with metabolic syndrome appear to respond as positively to the BWRP as those without metabolic syndrome. This view is further supported by the results of the model of multiple regression, which shows that ΔBMI, ΔFSS, and ΔSCT time are not associated with metabolic syndrome considered as an independent variable. An (expected) exception is represented by the association of metabolic syndrome with ΔCHD score, which is calculated by using some of the most important criteria (or risk factors) included in the definition of metabolic syndrome [21,22].

In the present study, ΔBMI was positively correlated with ΔCHD score, ΔFSS, and ΔSCT time. Furthermore, these three correlations were maintained throughout the analysis of all data from either the obese population with metabolic syndrome or the obese population without metabolic syndrome. This means that losing more weight results in an increasingly better cardiometabolic profile, muscle performance, and psychological well-being independently from having or not having metabolic syndrome. Therefore, any effort should be made to maximize the effectiveness of any BWRP, starting from a “rehabilitative personalization” for the unresponsive or scarcely responsive obese subjects such as obese females and older obese subjects.

Before closing, some limitations related to the experimental design of retrospective cohort studies such as ours should be mentioned: (1) the results obtained in retrospective studies are characterized by an inferior level of evidence compared with those obtained in the prospective studies, particularly randomized, placebo-controlled, double-blind and parallel-group studies, which are difficult to carry out in this research field; (2) our control group is represented by the same obese subject before BWRP, and this “obligated” choice might have spuriously incremented the effectiveness of the treatment, i.e., the 3-week BWRP; (3) retrospective studies are prone to recall bias or misclassification bias, including the so-called confounding effect, i.e., the possibility that other risk factors may be present, but not measured. So, caution should be adopted before any extrapolation of the results of the present study in a different context.

In conclusion, when comparing obese subjects undergoing a BWRP, metabolic syndrome is not a negative predictive factor affecting the effectiveness of this intervention in terms of weight loss, muscle performance, and psychological well-being. Paradoxically, the post-BWRP cardiometabolic profile and the ensuing CHD risk seem to be better in obese subjects with metabolic syndrome rather than without metabolic syndrome. These findings are valid even when sex-, BMI-, and age-related stratification of the obese population with or without metabolic syndrome is applied. Any effort should be made to promote weight loss because BWRP-induced benefits increase with weight loss and can be obtained in all obese subjects independently of the coexistence of metabolic syndrome.

## Figures and Tables

**Table 1:** Demographic, biochemical, and clinical characteristics of all obese subjects recruited in the study, subdivided into the two groups with or without metabolic syndrome, who underwent the 3-week BWRP.

All Obese Subjects						
Parameter	Without Metabolic Syndrome			With Metabolic Syndrome		
	Before	After	Δ (%)	Before	After	Δ (%)
N.	726	-	-	1196	-	-
Age (yr)	46.1 ± 15.3 ^a^	-	-	53.4 ± 12.6	-	-
BMI (kg/m^2^)	42.5 ± 5.3 ^a^	40.7 ± 5.1	−4.4 ± 1.4	43.8 ± 6.6	41.9 ± 6.4 ^b^	−4.3 ± 1.6
TOT-CHOL (mg/dL)	191.2 ± 35.5 ^a^	168.0 ± 31.1 ^b^	−11.4 ± 11.4 ^a^	198.0 ± 37.9	170.3 ± 35.0 ^b^	−13.1 ± 14.0
HDL-CHOL (mg/dL)	56.0 ± 12.2 ^a^	46.5 ± 10.4 ^b^	−16.0 ± 12.2 ^a^	45.6 ± 11.4	39.8 ± 9.3 ^b^	−11.6 ± 13.3
Glucose (mg/dL)	83.5 ± 11.9 ^a^	-	-	108.0 ± 36.9	-	-
Triglycerides (mg/dL)	99.9 ± 32.3 ^a^	-	-	159.4 ± 70.5	-	-
DBP (mmHg)	76.2 ± 7.9 ^a^	73.8 ± 6.4 ^b^	−2.3±11.3	77.6 ± 7.7	74.8 ± 6.6 ^b^	−2.8 ± 11.3
SBP (mmHg)	125.5 ± 13.8 ^a^	119.9 ± 9.0 ^b^	−3.7 ± 9.0 ^a^	130.4 ± 13.6	122.2 ± 9.7 ^b^	−5.6 ± 9.6
CHD score	3.1 ± 5.4 ^a^	3.3 ± 5.6	6.6 ± 102.3 ^a^	8.5 ± 4.8	7.9 ± 5.1 ^b^	−7.7 ± 79.2
FSS	34.5 ± 12.7 ^a^	26.9 ± 11.5 ^b^	−20.8 ± 20.2	38.3 ± 12.9	29.8 ± 11.8 ^b^	−20.8 ± 19.9
SCT (s)	5.7 ± 1.8 ^a^	5.4 ± 1.8 ^b^	−5.5 ± 6.1	6.4 ± 1.9	6.1 ± 1.9 ^b^	−5.2 ± 5.2

Abbreviations: BMI, body mass index; TOT-CHOL, total cholesterol; HDL-chol, high-density lipoprotein cholesterol; DBP, diastolic blood pressure; SBP, systolic blood pressure; CHD, coronary heart disease; FSS, fatigue severity score; SCT, stair climbing test. ^a^: *p* < 0.05 vs. group with metabolic syndrome; ^b^: *p* < 0.05 vs. the corresponding group before BWRP.

**Table 2:** Demographic, biochemical, and clinical characteristics of female and male obese subjects recruited in the study, subdivided into the two groups with or without metabolic syndrome, who underwent the 3-week BWRP.

	**Female Obese Subjects**			
**Parameter**	**Without Metabolic Syndrome**		**With Metabolic Syndrome**	
	**Before**	**After**	**Before**	**After**
N.	665	-	1035	-
Age (yr)	46.3 ± 15.3 ^a^	-	54.1 ± 12.2	-
BMI (kg/m^2^)	42.6 ± 5.3 ^a^	40.8 ± 5.1 ^b^	43.9 ± 6.6	42.1 ± 6.4 ^b^
TOT-CHOL (mg/dL)	191.9 ± 35.6 ^a^	169.5 ± 31.3 ^b^	198.6 ± 38.4	172.2 ± 35.4 ^b^
HDL-CHOL (mg/dL)	56.8 ± 12.1 ^a^	47.1 ± 10.3 ^b^	46.6 ± 11.4	40.4 ± 9.3 ^b^
DBP (mmHg)	76.0 ± 7.7 ^a^	73.8 ± 6.4 ^b^	77.5 ± 7.4	74.7 ± 6.5 ^b^
SBP (mmHg)	125.0 ± 13.4 ^a^	119.8 ± 8.9 ^b^	130.2 ± 13.5	122.3 ± 9.7 ^b^
CHD score	3.0 ± 5.6 ^a^	3.3 ± 5.8 ^b^	8.8 ± 4.9	8.4 ± 5.1 ^b^
FSS	34.9 ± 12.8 ^a^	27.1 ± 11.7 ^b^	39.2 ± 12.8	30.3 ± 12.0 ^b^
SCT (s)	5.7 ± 1.8 ^a^	5.4 ± 1.8 ^b^	6.5 ± 1.9	6.2 ± 1.9 ^b^
	**MALE Obese Subjects**			
**Parameter**	**Without Metabolic Syndrome**		**With Metabolic Syndrome**	
	**Before**	**After**	**Before**	**After**
N.	61	-	161	-
Age (yr)	44.1 ± 15.8 ^a^	-	48.7 ± 14.0	-
BMI (kg/m^2^)	41.9 ± 5.4	39.8 ± 5.2 ^b^	43.2 ± 6.5	41.0 ± 6.3 ^b^
TOT-CHOL (mg/dL)	183.4 ± 33.8 ^a^	152.0 ± 22.7 ^b^	193.9 ± 35.0	158.2 ± 29.5 ^b^
HDL-CHOL (mg/dL)	46.9 ± 10.2^a^	40.4 ± 9.6 ^b^	39.5 ± 9.2	35.6 ± 8.0 ^b^
DBP (mmHg)	78.7 ± 9.7	74.7 ± 6.6 ^b^	78.0 ± 9.5	75.2 ± 7.0 ^b^
SBP (mmHg)	131.1 ± 17.2	121.3 ± 10.1 ^b^	131.8 ± 14.2	121.8 ± 9.5 ^b^
CHD score	4.3 ± 3.0 ^a^	2.9 ± 3.6 ^b^	6.3 ± 3.3	4.4 ± 3.7 ^b^
FSS	30.1 ± 11.3	24.9 ± 9.8 ^b^	32.4 ± 12.4	26.8 ± 10.3 ^b^
SCT (s)	5.5 ± 1.6	5.2 ± 1.5 ^b^	5.7 ± 1.7	5.4 ± 1.6 ^b^

Abbreviations: BMI, body mass index; TOT-CHOL, total cholesterol; HDL-chol, high-density lipoprotein cholesterol; DBP, diastolic blood pressure; SBP, systolic blood pressure; CHD, coronary heart disease; FSS, fatigue severity score; SCT, stair climbing test. ^a^: *p* < 0.05 vs. group with metabolic syndrome; ^b^: *p* < 0.05 vs. the corresponding group before BWRP.

**Table 3:** Demographic, biochemical, and clinical characteristics of obese subjects with BMI < and ≥40 kg/m^2^ (obesity of degree 1–2 and degree 3, respectively), recruited in the study, subdivided into the two groups with or without metabolic syndrome, who underwent the 3-week BWRP.

	**Obese Subjects with BMI < 40 kg/m^2^**			
**Parameter**	**Without Metabolic Syndrome**		**With Metabolic Syndrome**	
	**Before**	**After**	**Before**	**After**
N.	247	-	360	-
Age (yr)	47.0 ± 15.3 ^a^	-	53.6 ± 13.7	-
BMI (kg/m^2^)	37.4 ± 1.9	35.8 ± 1.9 ^b^	37.5 ± 1.9	35.9 ± 1.9 ^b^
TOT-CHOL (mg/dL)	193.1 ± 36.8 ^a^	169.2 ± 32.1 ^b^	199.4 ± 37.4	170.3 ± 35.0 ^b^
HDL-CHOL (mg/dL)	56.7 ± 11.6 ^a^	48.2 ± 10.0 ^b^	45.2 ± 10.9	40.4 ± 9.3 ^b^
DBP (mmHg)	74.2 ± 7.3 ^a^	73.3 ± 6.4 ^b^	76.2 ± 7.4	73.7 ± 6.1 ^b^
SBP (mmHg)	124.0 ± 12.7 ^a^	119.3 ± 9.1 ^b^	128.2 ± 12.7	120.8 ± 9.0 ^b^
CHD score	3.1 ± 5.4 ^a^	3.2 ± 5.5	8.3 ± 5.2	7.5 ± 5.8 ^b^
FSS	32.3 ± 11.9 ^a^	25.7 ± 11.0 ^b^	36.6 ± 12.8	28.5 ± 11.1 ^b^
SCT (s)	5.3 ± 1.6 ^a^	5.1 ± 1.6 ^b^	6.0 ± 1.9	5.7 ± 1.8 ^b^
	**Obese Subjects with BMI ≥ 40 kg/m^2^**			
**Parameter**	**Without Metabolic Syndrome**		**With Metabolic Syndrome**	
	**Before**	**After**	**Before**	**After**
N.	479	-	836	-
Age (yr)	45.6 ± 15.3 ^a^	-	53.2 ± 12.1	-
BMI (kg/m^2^)	45.2 ± 4.6 ^a^	43.2 ± 4.4 ^b^	46.6 ± 6.0	44.5 ± 5.8 ^b^
TOT-CHOL (mg/dL)	198.3 ± 34.8 ^a^	167.4 ± 30.5 ^b^	197.4 ± 38.2	170.4 ± 35.0 ^b^
HDL-CHOL (mg/dL)	55.6 ± 12.6 ^a^	45.7 ± 10.5 ^b^	45.8 ± 11.6	39.5 ± 9.3 ^b^
DBP (mmHg)	77.2 ± 8.0 ^a^	74.1 ± 6.4 ^b^	78.2 ± 7.8	75.3 ± 6.7 ^b^
SBP (mmHg)	126.3 ± 14.4 ^a^	120.3 ± 8.9 ^b^	131.4 ± 13.8	122.8 ± 9.9 ^b^
CHD score	3.2 ± 5.5 ^a^	3.3 ± 5.6	8.5 ± 4.6	8.0 ± 4.9 ^b^
FSS	35.6 ± 13.0 ^a^	27.5 ± 11.8 ^b^	39.0 ± 12.9	30.4 ± 12.1 ^b^
SCT (s)	5.9 ± 1.9 ^a^	5.6 ± 1.8 ^b^	6.6 ± 1.9	6.2 ± 1.9 ^b^

Abbreviations: BMI, body mass index; TOT-CHOL, total cholesterol; HDL-chol, high-density lipoprotein cholesterol; DBP, diastolic blood pressure; SBP, systolic blood pressure; CHD, coronary heart disease; FSS, fatigue severity score; SCT, stair climbing test. ^a^: *p* < 0.05 vs. group with metabolic syndrome; ^b^: *p* < 0.05 vs. the corresponding group before BWRP.

**Table 4:** Demographic, biochemical, and clinical characteristics of <65 and ≥65 yr old obese subjects (non-older and older subjects, respectively), recruited in the study, subdivided into the two groups with or without metabolic syndrome, who underwent the 3-week BWRP.

	**<65 yr old Obese Subjects**			
**Parameter**	**Without Metabolic Syndrome**		**With Metabolic Syndrome**	
	**Before**	**After**	**Before**	**After**
N.	630	-	964	-
Age (yr)	42.4 ± 12.9 ^a^	-	49.5 ± 10.8	-
BMI (kg/m^2^)	42.6 ± 5.3 ^a^	40.7 ± 5.1 ^b^	44.2 ± 6.8	42.2 ± 6.5 ^b^
TOT-CHOL (mg/dL)	190.5 ± 35.6 ^a^	167.8 ± 31.0 ^b^	197.9 ± 38.0	170.9 ± 34.7 ^b^
HDL-CHOL (mg/dL)	55.1 ± 12.4 ^a^	46.2 ± 10.5 ^b^	45.1 ± 11.2	39.5 ± 9.2 ^b^
DBP (mmHg)	75.9 ± 7.7 ^a^	73.7 ± 6.5 ^b^	77.7 ± 7.6	74.8 ± 6.6 ^b^
SBP (mmHg)	124.0 ± 12.5 ^a^	119.4 ± 8.8 ^b^	129.9 ± 13.3	121.7 ± 9.4 ^b^
CHD score	2.3 ± 5.4 ^a^	2.5 ± 5.6	7.8 ± 5.0	7.1 ± 5.3 ^b^
FSS	33.2 ± 12.2 ^a^	25.4 ± 10.6 ^b^	37.4 ± 12.3	28.6 ± 11.2 ^b^
SCT (s)	5.5 ± 1.6 ^a^	5.2 ± 1.5 ^b^	6.2 ± 1.8	5.8 ± 1.7 ^b^
	**≥65 yr old Obese Subjects**			
**Parameter**	**Without Metabolic Syndrome**		**With Metabolic Syndrome**	
	**Before**	**After**	**Before**	**After**
N.	96	-	232	-
Age (yr)	70.3 ± 4.1	-	69.5 ± 3.9	-
BMI (kg/m^2^)	42.0 ± 5.5	40.3 ± 5.3 ^b^	42.4 ± 5.7	40.8 ± 5.5 ^b^
TOT-CHOL (mg/dL)	196.2 ± 34.4	169.3 ± 31.8 ^b^	198.3 ± 37.8	168.0 ± 36.3 ^b^
HDL-CHOL (mg/dL)	61.8 ± 9.3 ^a^	49.1 ± 9.4 ^b^	47.9 ± 11.7	40.9 ± 9.8 ^b^
DBP (mmHg)	77.9 ± 9.0	74.6 ± 6.0 ^b^	77.2 ± 8.2	74.6 ± 6.6 ^b^
SBP (mmHg)	135.1 ± 17.8	123.6 ± 9.6^b^	132.6 ± 14.6	124.4 ± 10.4 ^b^
CHD score	7.6 ± 2.4 ^a^	7.8 ± 2.6	10.9 ± 3.1	10.6 ± 3.3
FSS	43.4 ± 12.7	36.8 ± 12.7 ^b^	41.7 ± 13.5	35.0 ± 12.9 ^b^
SCT (s)	7.9 ± 2.1	7.6 ± 2.0 ^b^	7.7 ± 2.0	7.4 ± 1.9 ^b^

Abbreviations: BMI, body mass index; TOT-CHOL, total cholesterol; HDL-chol, high-density lipoprotein cholesterol; DBP, diastolic blood pressure; SBP, systolic blood pressure; CHD, coronary heart disease; FSS, fatigue severity score; SCT, stair climbing test. ^a^: *p* < 0.05 vs. group with metabolic syndrome; ^b^: *p* < 0.05 vs. the corresponding group before BWRP.

## Abbreviations

BMI, body mass index; CHD, coronary heart disease; CV, coefficient of variation; DBP, diastolic blood pressure; FSS, fatigue severity score; HDL, high-density lipoprotein; SCT, stair climbing test; sd, standard deviation; SBP, systolic blood pressure; TOT, total; yr, year.
